# Familial Glucocorticoid Deficiency Presenting with Tonic-Clonic Seizure: A Case Report

**DOI:** 10.3390/children10020301

**Published:** 2023-02-03

**Authors:** Ahmed Hassan Alghamdi

**Affiliations:** Department of Pediatrics, Al Baha Medical College, Al Baha University, Al Baha 65779-7738, Saudi Arabia; ahsaeed@bu.edu.sa

**Keywords:** familial glucocorticoid deficiency, seizures, hypoglycemia, hyperpigmentation

## Abstract

**Highlights:**

- Familial glucocorticoid deficiency is a rare disease with nonspecific clinical presentations that can lead to late diagnosis with high rates of morbidity and mortality.
- In a child with features of low serum cortisol and high ACTH, namely, hypoglycemia and hyperpigmentation, FGD should have a place in the differential diagnosis list.

**Abstract:**

Introduction: Familial glucocorticoid deficiency (FGD) is a rare cause of adrenal insufficiency in children. The condition can present with features of low cortisol and high adrenocorticotropic hormone (ACTH). Late diagnosis can be associated with high morbidity and high mortality rates. Patient: The presented case was a three-year-old Saudi girl who presented with dehydration and seizures as a complication of hypoglycemia. The initial examination and investigations revealed hyperpigmentation and normal arterial blood pressure. The ***lab investigation and genetic study*** revealed hypoglycemia, metabolic acidosis, low serum cortisol: 53 nmol/L (N: 140–690 nmol/L), normal androgens: 0.65 nmol/L (N: 0.5–2.4 nmol/L) and aldosterone: 50 pgmL (N: 2–200 pg/mol), and normal serum electrolytes. The ACTH level was more than 2000 pg/mL. A genetic study indicated a homozygous likely variant in the nicotinamide nucleotide transhydrogenase (*NNT*) gene, consistent with a genetic diagnosis of autosomal recessive glucocorticoid deficiency type 4. No mutations were found regarding MC2R, MRAP, and TXNRD2. Intervention and outcome: The child was started on hydrocortisone, initially at 100 mg/m^2^/dose IV and then 100 mg/m^2^/day divided to q 6 hr. The dose was gradually decreased to 15 mg/m^2^/day PO BID, with clinical improvement and normalization of the serum ACTH level. Conclusions: The autosomal recessive glucocorticoid deficiency, a variant of FGD type 4, is a very rare condition that may lead to high rates of mortality when the diagnosis and treatment occur late. Therefore, early diagnosis and treatment is essential for good outcomes.

## 1. Introduction

Familial glucocorticoid deficiency (FGD) is an uncommon autosomal recessive disorder in which there is deficient cortisol and androgen secretions and ACTH stimulation unresponsiveness, so ACTH resistance or adrenal unresponsiveness to ACTH is the alternative name for this disorder.

Serum cortisol concentrations are low or undetectable and plasma ACTH concentrations are high. Aldosterone secretion is usually normal and responds to volume depletion and postural stimuli. The disorder usually presents in childhood with hyperpigmentation, muscle weakness, hypoglycemia, and seizures, and it may be associated with achalasia and absent lacrimation (Allgrove syndrome or triple A syndrome) [1]. The incidence of FGD is unknown; reported cases of FGD around the world are very limited [2,3,4].

Microscopical examination of adrenal gland revealed marked atrophic changes in both zona fasciculata and zona reticularis, while no abnormalities were observed in zona glomerulosa [5]. This leads to a low level of the plasma cortisol concentration, which is reflected in the marked elevation of ACTH levels owing to the lack of negative feedback of the hypothalamus. On the other hand, androgen, aldosterone, and plasma renin are within the normal reference range [6,7].

According to the marked elevation of ACTH, the initial feature to appear is skin and mucus membrane hyperpigmentation owing to melanin overproduction and advanced bone age. The patient is highly prone to developing nonketotic, nonhyperinsulinemic hypoglycemia, especially on exposure to stress or infection [8].

The initial symptoms may appear early during the neonatal period in the form of feeding difficulties, regurgitation, failure to increase weight, and recurrent infection and hypoglycemia, which if untreated can lead to recurrent seizures, developmental delay, learning difficulties, neurological damage as spastic quadriplegia and severe mental disability, and even death [9].

In addition, low serum cortisol may lead to generalized weakness, weight loss, fatigue, anorexia, nausea, vomiting, altered bowel habits as attacks of unexplained diarrhea and constipation, flank and abdominal pain, and hypothermia [10].

Other children may be presented by absent adrenarche and tall stature. Consanguinity, positive family history, and unexplained neonatal death support the FGD diagnosis.

The differential diagnosis of FGD includes disorders presented by adrenal insufficiency as Allgrove syndrome (AAA syndrome), characterized by alacrimia, achalasia of the esophageal cardia, adrenal failure, congenital adrenal hyperplasia (CAH), adrenoleukodystrophy, pediatric hypopituitarism, and confirm the FGD diagnosis, normal very long chain fatty acids, and serum electrolytes, will confirm the FGD diagnosis [11,12].

Radiological scanning is very important for diagnosis. The adrenal gland in FGD is small sized, while in other disorders the gland shows enlargement, as seen in infiltrative and storage disease. In addition, the scanning will exclude other disorders associated with calcification such as tuberculosis [13].

## 2. Patient Information

A three-year-old Saudi girl was admitted to the emergency department with a present history of abnormal movement, fever, and cough for the preceding two days. The patient’s parents did not detect any abnormal general health condition until the child started to present with intermitted cough and fever. The patient went to primary health care and received an antipyretic and antibiotic, but the fever did not improve. Two days later, the patient exhibited abnormal movements of both the upper and lower limbs in the form of tonic-clonic seizures with up-rolling of the eyes. There was no positive history regarding the other symptoms, no history of drug intake or injection, no history of trauma, no dysphagia or repeated vomiting, and no history of diabetes mellitus, neurological, cardiac, renal, hepatic or any chronic medical conditions, apart from a positive history of two hospital admissions with febrile convulsion, at a 5 month interval, and diagnosed as bronchial asthma and bronchopneumonia. The mother noticed a generalized blackish coloration of his skin at the age of 18 months.

Natal history revealed that the child was full-term, normal vaginal delivery, appropriate for gestational age, no history of prenatal or natal asphyxia or manifestations of neonatal distress, intact neonatal reflexes, and a high APGAR score. There is a positive history of consanguineous parents. There is a positive family history of one brother who presented at the age of 6 month with severe dehydration, shock, hyperpigmentation, increased serum ACTH level, low level of cortisol and normal level of androgens and aldosterone. The case was clinically diagnosed with congenital adrenal hyperplasia or primary adrenal insufficiency and on hydrocortisone since the age of two months, but a genetic confirmation of the diagnosis was not conducted for the brother. No family history for tuberculosis infection or contact with tuberculous patient. Both parents are secondary educated and of middle-income and middle-socioeconomic standards, and they were not enthusiastic regarding conducting genetic testing for them and the affected brother. No genetic analyses were performed for both parents.

## 3. Clinical Findings

On examination, no dysmorphic features were noticed. The heart rate was 130/min, the arterial blood pressure was 110/60 mmHg, the respiratory rate was 40/min, the body temperature was 38.3 °C, and the patient had hyperpigmentation all over the body, more marked on both elbows, hands, knees, and buccal mucosa. A neurological examination revealed hyporeflexia and hypotonia. The examination of the genitalia revealed no abnormalities, with normal lacrimation. No signs of achalasia were noticed. No other abnormal findings were detected.

## 4. Diagnostic Assessment and Therapeutic Intervention

At this stage, the patient was found to be hypoglycemic (random blood glucose of 30 mg/dL). She received IV dextrose 10% bolus 2 mL/kg, then IV dextrose 10%/half normal saline maintenance 1.5 mL/kg, until she improved and started good oral intake.

On further investigation, an arterial blood gas (ABG) test revealed a pH of 7.24, HCO_3_ of 14 mEq/L, PaCO_3_ 45 mmHg, and the sodium, potassium, and chloride were normal. The serum calcium, phosphorous, and magnesium were normal. The serum albumin, AST, ALT, alkaline phosphatase, blood urea nitrogen, creatinine, glycated hemoglobin, free T_3_, free T_4_ and TSH, serum insulin, and C-peptide were all found to be normal. The 17-hydroxyprogesterone was normal. The ACTH was more than 2000 pg/mL in a critical blood sample (with hypoglycemia). Low serum cortisol: 53 nmol/L (N. 140–690 nmol/L) and normal androgens: 0.65 nmol/L (N. 0.5–2.4 nmol/L) and aldosterone: 50 pgmL (N. 2–200 pg/mol). The sonographic appearance showed that both his adrenal glands were very small; his right adrenal was measured 5 × 6 mm and left adrenal was 5 × 7.5 mm in diameter. Computerized tomography of CNS was normal. Therefore, a diagnosis of FGD was suspected, and a general genetic study confirmed the diagnosis.

After the discovery of the signs of adrenal insufficiency, the patient was started on hydrocortisone, initially IV 100 mg/m^2^/dose and then 100 mg/m^2^/day divided to q 6 hr. The dose was gradually decreased to 15 mg/m^2^/day P OBID. At follow-up, the patient showed improvement of the symptoms and laboratory investigations indicated the ACTH to be normalized to 9.30 pg/mL without recurrence of further hypoglycemic attacks.

## 5. Genetic Study

The interpretation of the genetic study showed a homozygous likely variant detected in the nicotinamide nucleotide transhydrogenase (*NNT*) gene. This result is in consistent with the diagnosis of genetic autosomal recessive glucocorticoid deficiency type 4, with or without mineralocorticoid deficiency. No mutations were found regarding MC2R, MRAP, and TXNRD2.

The genomic DNA was fragmented by an enzymatic assay, and the regions of interest were supplemented through capture probes for DNA. These specific regions consist of approximately fortyone Mb of coding exome of human (targeting is more than ninety-eight percent 98% of the coding reference sequence (RefSeq) of the build of human genome GRCh37/hg19), in addition to genome of the mitochondria. The generated reference library was structured on the Illumina platform to achieve at least 20x coverage depth for more than ninety-eight percent of the targeted bases. The in-house bioinformatics channel, together with read orientation to GRCh37/hg 19 genome revised Cambridge Reference Sequence (rCRS) of the mitochondrial DNA of human (NC_012920), annotation, variant calling, and comprehensive variant filtering was utilized. The whole variants having a minor allele frequency (MAF) of less than one percent in the AD database genome and the disease-causing variants that was reported in the HGMD®, ClinVar, or CentoMD® were deeply re-evaluated. The research for the relevant variants was concentrated on the coding exons and flanking plus/minus intronic genes nucleotides with clear gene-phenotype proof (according to information obtained from the OMIM^®^). The whole possible patterns for the inheritance mode were put in consideration. Furthermore, the obtained family history in addition to clinical information were utilized to assess the identified variants involving their causality and pathogenicity. The variants were categorized into main five classes (the pathogenic, likely pathogenic, variant of uncertain significance, likely benign, and benign) alongside the ACMG guidelines for variants classification. 

The whole relevant variants linked to the patient phenotype were stated. Variants of low feature in addition to unclear zygosity were proved by the orthogonal method. Subsequently, a specificity of more than 99.9% for all registered variants was justified. Variants of Mitochondria were registered for heteroplasmy levels of fifteen percent or more. The copy number variant (CNV) revealing software has a sensitivity of more than ninety-five percent for all zygous (homo/hemi) and mitochondrial deletions, in addition to heterozygous deletions, hemizygous duplication spanning at least three consecutive exons and duplications of homozygous. For the whole the uniparental disomy (UPD) screening, a specific set of rules was used to evaluate the well-identified chromosomal regions (6q24, 7, 11p15.5, 14q32, 15q11q13, 20q13, and 20) that were clinically relevant [14]. 

Variant interpretation. *NNT* variant c.2877-2A is expected to interrupt the highly preserved acceptor splice site. It is categorized as likely pathogenic (class 2) corresponding to the recommendations applied by CENTOGENE and ACMG [14,15]. ACMG recommendations for variant classification:

- Class 1 called “Pathogenic” variant.
- Class 2 called “Likely pathogenic.”
- Class 3 called “Variant of uncertain significance (VUS)”
- Class 4 called “Likely benign.”
- Class 5 called “Benign.”

In addition, other varieties of the clinically relevant variants can be recognized (e.g., risk factors, some modifiers). The pathogenic variants in the *NNT* gene cause autosomal recessive glucocorticoid deficiency type 4, with or without mineralocorticoid deficiency (GCCD4, OMIM^®^: 614736) [16]. We did not detect any class 1 or 2 variants in the genes, for which incidental findings are to be identified.

## 6. Discussion

The most common causes of primary adrenal insufficiency are autoimmune disorders and, to a lesser degree, drugs, infections, and metastatic disease. Some other less common disorders can also cause adrenal insufficiency. Familial glucocorticoid deficiency usually presents in childhood, with the complications of cortisol deficiency and ACTH excess. Our patient presented with features suggestive of adrenal insufficiency, including hypoglycemia, metabolic acidosis, and hyperpigmentation. The early identification of these features is important, as the disease may lead to complications, including learning difficulties, spastic paraplegia, and stunted growth, as reported in an Ethiopian infant who presented with severe hypoglycemic attacks leading to spastic quadriparesis, psychomotor retardation, and microcephaly [17]. Familial glucocorticoid deficiency may even be fatal if treatment is not commenced properly.

Approximately 25% of patients with isolated FGD have mutations of the melanocortin-2 (ACTH or MC2R) receptors [18]. Affected individuals may be homozygotes or compound heterozygotes for point mutations that alter a critical amino acid, alter the reading frame, or truncate the ACTH receptor [19]. Patients with MC2R mutations are considered to have FGD type 1.

Up to 20% of patients have MRAP gene mutation (melanocortin-2 receptor accessory protein) without mutations of the promoter or coding region or of the receptor of MC2R gene [20,21]. The interaction of this protein with the MC2R may play a role in trafficking the MC2R from its normal pathway to locate in the cellular surface. These patients are considered to have FGD type 2. So, according to the presence or absence of mutation in ACTH receptor, FGD is categorized into type 1 (with ACTH receptor mutations) and type 2 (with normal ACTH receptors) [22,23]. 

In 2009, low number of patients were revealed to have STAR mutations [24], producing congenital adrenal hyperplasia of lipoid type (LCAH; FDG type 3), in which severe adrenal and gonadal insufficiency were described. In addition, in 2011, a few patients among the Irish traveller community who is genetically isolated were discovered to carry out mutations in MCM4 (the mini chromosome maintenance protein 4). These cases represented for nearly one percent of the FGD cohort [25].

Most recently, 10% of cases were found to have mutations in nicotinamide nucleotide transhydrogenase (*NNT*) which has other alternative names as or energy-linked transhydrogenase or NADP transhydrogenase or NADPH transferase or pyridine nucleotide transhydrogenase, or GCCD4. This enzyme is embedded along the inner mitochondrial membrane and helps in producing NADPH, responsible for removing the potentially toxic reactive oxygen species and, thus, preventing DNA damage and helping DNA repair program. In addition, *NNT* help to maintain the integrity of intracellular proteins and cell membranes. *NNT* is abundant in the adrenal and thyroid glands, as well as kidneys, heart, and fatty tissue. The mutation of the *NNT* gene impairs the production of NADPH, leading to an accumulation of the reactive oxygen species in adrenal and thyroid glands and other tissues, leading to the development of adrenal dysfunction and FGD type 4. It is vaguely understood why *NNT* gene mutations seem to select only adrenal and thyroid glands. Patients with *NNT* mutations have been found with a clinical manifestation of congenital hypothyroidism owing to the development of thyroid dysgenesis [26,27].

As mentioned above, *NNT* is represented in all human cells, and mutations of this gene could give rise to other symptoms in addition to FGD, as reactive oxygen species produce cellular and molecular harmful effects.

FGD type 4 account for about 10% of the FGD cohort [17]. Approximately forty percent of the cased diagnosed as FGD still have an unclear etiology. The genetic study of our patient revealed a homozygous likely variant determined in the *NNT* gene, that is in consistent with the diagnosis of autosomal recessive FGD type 4. Sequencing of one hundred FGD patients of unclear etiology showed about 23 compound or homozygous or heterozygous mutations in appeared in 16 kindreds, representing for ten percent of the FGD cohort. The mutations covered the twenty-two exons of *NNT*, and varied from missense, nonsense mutations, to splice site mutations and yet the abolition of the initial methionine [17,27]. Analysis into the mechanism of disease appeared that *NNT* is broadly expressed in human tissues mainly the heart, adrenal, kidney, adipose tissue, and thyroid [17,27]. Replacement with hydrocortisone is expected to prevent further hypoglycemic bouts and suppress ACTH levels and is therefore the recommended treatment for FGD. The suppression of plasma ACTH levels in FGD can be very difficult, but this target was achieved in our case.

Replacement therapy by hydrocortisone is the primary goal of the treatment of FGD not through suppression of elevated ACTH, which is very difficult in certain conditions. Taking into consideration the dose of 10 to 12 mg/m^2^/day divided into regular three oral doses is usually sufficient. Parents should be educated regarding the conditions that require higher hydrocortisone dosages, such as in acute illness and emergency conditions [28]. No restriction for live attenuated vaccines was recommended [28]. Physical interest and activity are required in effectively treated cases. The FGD is a rare disease and must be in differential diagnosis with cases presented by frequent hypoglycemia, repeated uncontrolled seizures, and unexplained infant death.

## 7. Conclusions

FGD should be considered in any presenting case with recurrent symptomatic hypoglycemia. The rapid diagnosis and early treatment will be lifesaving. Parental education concerning increasing hydrocortisone is crucial, especially in stress and emergency conditions. Genetic counseling must be performed for any suspected cases, particularly those with a positive family history. Cases diagnosed as FGD type 4 (*NNT* mutation) should be scanned for other abnormalities, especially thyroid gland hypofunction. More research should be conducted to detect other associations with FGD type 4.

## Data Availability

All data supporting results can be obtained from the corresponding author of the work through the email: ahsaeed@bu.edu.sa.
